# Real world evaluation of three models of NHS smoking cessation service in England

**DOI:** 10.1186/1756-0500-5-9

**Published:** 2012-01-06

**Authors:** Tony Mardle, Shirley Merrett, Jane Wright, Fran Percival, Ian Lockhart

**Affiliations:** 1 South East Essex PCT, South Essex Stop Smoking Service, Mapline House, 14 Bull Lane, Rayleigh, Essex SS6 8JD, UK; 2 Formerly of: Bournemouth and Poole Teaching PCT, Canford House, Discovery Court Business Centre, 551-553 Wallisdown Road, Poole, Dorset BH12 5AG, UK; 3 Warwickshire PCT, Westgate House, Market Street, Warwick CV34 4DE, UK; 4 pH Associates, Derwent House, Dedmere Road, Marlow SL7 1PG, UK; 5 Pfizer Ltd, Walton Oaks, Dorking Road, Tadworth, Surrey KT20 7NS, UK

**Keywords:** Smoking cessation, Service structure, Service development, Nicotine replacement therapy, Varenicline, Bupropion

## Abstract

**Background:**

NHS Stop Smoking Services provide various options for support and counselling. Most services have evolved to suit local needs without any retrospective evaluation of their efficiency.

Three local service evaluations were carried out at Bournemouth & Poole Teaching Primary Care Trust (PCT) (PCT1), NHS South East Essex (PCT2) and NHS Warwickshire (PCT3) to describe the structure and outcomes associated with different services.

**Result:**

Standardised interviews with key personnel in addition to analysis of data from 400 clients accessing the service after 1^st ^April 2008 in each PCT. The PCTs varied in geography, population size and quit rate (47%-63%). Services were delivered by PCT-led specialist teams (PCT1), community-based healthcare providers (PCT3) and a combination of the two (PCT2) with varying resources and interventions in each.

Group support resulted in the highest quit rates (64.3% for closed groups v 42.6% for one-to-one support (PCT1)). Quit rates were higher for PCT (75.0%) v GP (62.0%) and pharmacist-delivered care (41.0%) where all existed in the same model (PCT2). The most-prescribed therapy was NRT (55.8%-65.0%), followed by varenicline (24.5%-34.3%), counselling alone (6.0%-7.8%) and bupropion (2.0%-4.0%).

**Conclusion:**

The results suggest that service structure, method of support, healthcare professional involved and pharmacotherapy all play a role in a successful quit. Services must be tailored to support individual needs with patient choice and access to varied services being key factors.

## Background

The effects of cigarette smoking on individual health [1], the health service [2] and the economy [3] are well-documented. In view of this wide-ranging impact, a previous UK government funded a comprehensive National Health Service (NHS) Stop Smoking Service with a target of reducing the number of adult smokers to 21% or fewer by 2010 [4]. The current Tobacco Control Plan for England includes clear goals to reduce adult smoking prevalence from 21% to 18.5%, smoking rates among 15 year olds from 15% to 12% and smoking in pregnancy from 14% to 11% by 2015 [5]. The NHS is free at the point of delivery and funded by taxation.

NHS Stop Smoking Services [4] are now available throughout the NHS in England, providing counselling, behavioural support and medications to smokers wanting to quit. Smokers accessing these services are given various options [4] for the type of support and counselling they wish to receive. While most smoking cessation services offer both one-to-one support and sessions run in groups, the latter may be either open/rolling (people joining and leaving all at different stages of the quit process) or closed (all participants at the same stage of the quit process) and in some cases may be delivered in large workplaces ('workplace groups'). Support may be delivered centrally by Primary Care Trust (PCT)-based smoking cessation advisors or may be commissioned from GPs, nurses and pharmacists in the community. The majority of services have evolved their structure over time, to suit local needs, without evaluating whether the service is operating in the best possible way. These services also offer patients access to pharmacological therapies including nicotine replacement therapy (NRT), bupropion and varenicline. In the UK, NRT can be purchased from pharmacies without a prescription, and so can be provided by nurses and pharmacists providing a smoking cessation service, while the other therapies are available only with a physician's prescription.

Data are routinely collected to allow the Department of Health to evaluate the Stop Smoking Service. Quit rates are reported by the NHS Information Centre [6] by gender, age, ethnic group, pregnancy and geographical area, but not by structure of the service, which differs significantly between PCTs.

This series of service evaluations describe the resources used and outcomes associated with differently-structured services within three PCTs: Bournemouth & Poole Teaching PCT (PCT1), NHS South East Essex (PCT2) and NHS Warwickshire (PCT3).

## Methods

Local service evaluations were conducted separately in three PCTs between January and September 2009 by an independent healthcare evaluation consultancy, using the same methods for each one. No approval was sought from a Research Ethics Committee nor consent from clients as it is required only for research and not service evaluations in the UK. Each Trust did however gain Trust management approval to take part in the evaluation and to release anonymised data. Locally and nationally published statistics [7-18] were used to obtain local population demographics so that the structure and outcomes of each service could be set in its local context.

A description of each service and the staff resources providing it was obtained by conducting interviews with smoking cessation service staff. The service manager was interviewed in all three PCTs to obtain information on the structure of the service, while a selection of key staff providing the service were asked for estimates of the average staff time involved in a quit attempt. Standardised interview guides for the service lead and for service representatives were used to ensure consistency between interviews, and responses were documented by the interviewer during the interview. Interviews were not audio or video recorded as the data collected was factual and was easily recorded onto a proforma.

Patient level data from each of the smoking cessation services were obtained retrospectively from 400 client records from each PCT. The first 400 clients accessing the service after 1st April 2008 were included in the review at each PCT. This represented 13.0%, 7.3% and 6.3% of all people from PCT1, PCT2 and PCT3 respectively that set a quit date from April 2008 to March 2009 with the NHS Stop Smoking Service [19]. Data were collected by PCT staff from electronic records and the DoH 'Gold standard' service monitoring forms. Anonymised-coded data on client demographics, chosen service provider and intervention and quit attempt outcomes were provided to the consultancy for analysis using Microsoft Excel™ and reporting back to each PCT.

## Results

The numbers and professions of the service provider staff interviewed varied according to the structure of the service described by the manager: in PCT1, three smoking cessation advisors, in PCT2, three advisors, four pharmacists and two practice nurses and in PCT3, three pharmacists and two practice nurses were interviewed.

### Description of service

PCT1 covers a population of 301,732 [20] in an area of just 42.9 square miles [21], where an estimated 20% [22,23] of people smoke and health is generally similar to the national average (Bournemouth) or above average (Poole). At the time of evaluation the smoking cessation service was being delivered by a PCT-led specialist smoking cessation team (See Table 1) by means of a choice of group and one-to-one support (Table 1). This smoking cessation service was accessed by 3076 people from April 2008 to March 2009 [23].

**Table 1:** Staffing structure of smoking cessation service in 3 PCTs

PCT	PCT1	PCT2	PCT3
Service model	PCT provided	Combination PCT & HCP provided	HCP provided

PCT Staffing	1 clinical specialist lead (1.0 whole time equivalents (WTE)) 3 administrators (3.0 WTE) 1 co-ordinator/advisor (1.0 WTE) 6 advisors (5.91 WTE) band 5/6 - core team 1 smoking and pregnancy advisor (0.8 WTE) 1 maternity support worker (0.2 WTE) 10 bank staff (total 0.5 WTE) 1 WTE Service Manger vacancy	1 Stop smoking service & tobacco control manager (1.0 WTE) 2 administrators (1.5 WTE) Stop smoking advisors (2.5 WTE) 1 smoking and pregnancy advisor (0.5 WTE) Primary care facilitators (1.5 WTE) 1 Secondary care stop smoking advisor (0.5 WTE) Trainers (2.0 WTE) Stop smoking sessional advisors, ad hoc to service need. (1.5 WTE)	1 Smoking Cessation (SC) Manager (1.0 WTE) 1 Data & Information Assistant (1.0 WTE) 1 Finance & Training Administrator (0.45 WTE) 5 Advisors (3.7 WTE) 3 Pregnancy SC Advisors (1.5 WTE) 1 Tobacco Control Co-ordinator (0.5 WTE) (Public Health)

Total PCT WTE	12.41	11.0	8.15

Other providers	None	GP Practices n=68 Pharmacies n=61 Dental practices n=3 Opticians n=2	GP practices n=76 Pharmacies n=49 Dental practices n=2

Total other providers	0	134	127

Intervention type:			

One to one	263 (65.7%)	211 (52.8%)	396 (99.0%)

Groups	133 (33.3%)	185 (46.3%)	0

Other (drop-in, telephone, family/couple)	4 (1.0%)	4 (1.0%)	4 (1.0%)

PCT2 covers a population of 325,354 [24] in an area of 98.1 sq miles [25], where approximately 21.4% [26] of people smoke and overall health is similar to (Southend-on-sea) or better than (Castle Point & Rochford) the English national average. The smoking cessation service is PCT-led by a specialist smoking cessation team but delivered by a combination of the PCT team and general practices, pharmacies, dentists and opticians (See Table 1) employing both one to one and group support (Table 1). The PCT2 smoking cessation service was accessed by 5497 people from April 2008 to March 2009 [27].

PCT3 covers a population of 505,860 [28] in an area of 736 square miles [29], where approximately 23% [30] of people smoke and people's health is generally better than the average for England, apart from Nuneaton and Bedworth where it is worse than average. The stop smoking service was delivered to 6348 people from April 2008 to March 2009 [31] by local healthcare professionals (Table 1). Support is primarily by one to one support (Table 1), rather than in groups. There is little or no direct contact with smokers by the centralised PCT team, whose main role is training and support of the service providers.

### Client access to support

In both PCT1 and PCT2, where clients can access either one to one or group support, the latter yielded the higher carbon monoxide (CO) validated four-week quit rates (54.9% and 76.2% for group support vs 42.6% and 51.2% for one to one support in PCT1 and PCT2 respectively; Table 2). However, in PCT3, where only one to one support is provided, a similar quit rate to the PCT1 group support quit rate was achieved (PCT3 one to one support 55.8% vs PCT1 group support 54.9%) (Table 2). Of the different types of group support offered in PCT1, the workplace groups produced the highest quit rate (though absolute numbers were small) of 64.3%, but an even higher quit rate was achieved with closed groups in PCT2 (76.2%) (Table 2). To put this into perspective, national figures for April 2008 to March 2009 give a quit rate of 49% for one to one support, 64% for closed groups and 55% for rolling groups [32].

**Table 2:** Four week (CO validated) quit rate by service provider and intervention type

	PCT1	PCT2	PCT3
**Service provider**			

PCT	46.8%	75.0%	-

GP	-	62.0%	58.2%

Pharmacist	-	41.0%	43.0%

**Intervention type**			

**One to one**	**112/263 (42.6%)**	**108/211 (51.2%)**	**221/396 (55.8%)**

Closed group	8/17 (47.1%)	141/185 (76.2%)	-

Rolling group	47/88 (53.4%)	-	-

Workplace group	18/28 (64.3%)	-	-

**Total - all groups**	**73/133 (54.9%)**	**141/185 (76.2%)**	-

Other (drop in, telephone, family/couple)	2/4 (50.0%)	4/4 (100.0%)	4/4 (100.0%)

**Overall 4-week quit rate**	**187/400 (46.8%)**	**253/400 (63.3%)**	**225/400 (56.3%)**

Where PCT and community delivered services exist within the same model of provision (i.e. PCT2; Table 1), four-week quit rates were higher within the PCT delivered services (75.0% quit rate for PCT care vs 62.0% quit rate for GP-delivered care and 41.0% quit rate for pharmacist-delivered care).

### Pharmacotherapy

In terms of prescriptions, all agents were available for first line use in each of the Trusts - there were no prescribing restrictions in place at the time of data collection. The most commonly used pharmacotherapy was nicotine replacement therapy (NRT) (PCT1, 65.0%; PCT2, 55.8%; PCT3, 63.5%), followed by varenicline (PCT1, 24.5%; PCT2, 34.3%; PCT3, 25.5%) and bupropion (PCT1, 2.8%; PCT2, 4.0%; PCT3, 2.0%). In PCT1, over half (56.9%) of those using NRT used combination NRT, as they were assessed as being complex clients with high nicotine addiction. This data was not available for the other PCTs. Within each PCT, a consistently small number of clients chose to attempt to quit with counselling alone (PCT1, 6.0%; PCT2, 5.8%; PCT3, 7.8%). These figures are largely in line with the national average during the year April 2008 to March 2009 where 67% of people who set a quit date were prescribed NRT, 20% varenicline, 2% bupropion and 5% opting for no therapy [33].

Quit rates associated with pharmacotherapy interventions are shown in Table 3. Quit rates associated with NRT ranged between 40.0% and 54.3%; between 60.2% and 81.0% for varenicline; and between 37.5% and 90.9% for bupropion, although numbers for the latter are small across all three PCTs. Quit rate in those opting to attempt to quit with counselling alone ranged from 35.5% to 56.5%. Pharmacological support provided varied across service providers (PCT, GP, pharmacist), with pharmacists tending to prescribe mainly NRT (100.0% of pharmacist prescribing in PCT2, 87.3% in PCT3) and the highest proportion of varenicline being prescribed by PCT employed smoking cessation advisors (56.0% of PCT prescribing was for varenicline versus 25.0% of GP prescribing within PCT2). Prescribing also differed across service delivery settings, with PCT2 showing more varenicline prescribing within the group setting (58.4% of clients setting a quit date within a group) than clients attempting to quit with one to one support (12.3%). Bupropion was rarely used (2.9% of quitters overall, 0-7.0%, depending on the provider).

**Table 3:** Four week quit rate by pharmacological intervention (denominator n = number clients setting quit date)

	PCT1	PCT2	PCT3
NRT	40.0% (104/260)*	52.0% (116/223)	54.3% (138/254)

Varenicline	60.2% (59/98)	81.0% (111/137)	68.6% (70/102)

Bupropion	90.9% (10/11)	75.0% (12/16)	37.5% (3/8)

None	41.6% (10/24)	56.5% (13/23)	35.5% (11/31)

*This result represents a combination of monotherapy (53/112; 47%) and dual therapy (51/148; 34%).

## Discussion

This retrospective evaluation of smoking cessation services in 3 English PCTs shows that the context of these services varies widely between PCTs. The general health of the populations in these 3 PCTs did not differ widely from the national average. However, the prevalence of smoking ranged from 20% to 23%. The area covered by the PCT ranged from just 43 square miles in PCT1 to 736 square miles in PCT3, and the populations served ranged from 300,000 to over 500,000. All these differences have informed the development of the varying models of smoking cessation service in order to meet the needs of the local population.

The lower rates of smoking prevalence in PCT1, which follow several years of declining prevalence, may suggest that those who are more motivated to quit have already done so. Hence the service provided in PCT1 by specialised stop smoking advisors, with a wide choice of different interventions is needed for continuing success with remaining smokers who may have more complex needs. This need for specialist advisors is likely to become increasingly important as prevalence rates continue to be driven down.

Conversely, in PCT3, which covers a large geographical area, it is not feasible to provide group support to the widely dispersed, predominantly rural population. As such, the service is appropriately composed almost exclusively of one to one support provided by GP practices and pharmacies, with emphasis on specialist advisors providing high quality training and support to those delivering the service.

The 4-week quit rates for all types of intervention were greater than 40.0%, with overall quit rates averaging 46.8% to 63.3% across the PCTs. Quit rates varied between types of intervention, ranging from 42.6% for one to one support to 64.3% for closed groups in PCT1.

The 4-week quit rates also varied between the PCTs for similar types of intervention; e.g. one to one support produced quit rates ranging from 42.6% (PCT1) to 55.8% (PCT3), while group support quit rates ranged from 47.1% (PCT1) to 76.2% (PCT2). The quit rates for one to one support are similar to the national average for 2010 (49%) [33]. However, in terms of group support, PCT1 had quit rates (54.9%) that were generally slightly lower and PCT2 had quit rates that were slightly higher (76.2%) than the national average (64%) [34].

The data suggest that, whilst group support can be an efficient and effective method, it does produce variable results which may depend on the type of client (e.g. more or less motivated), staff and geographical setting of the service. These factors need to be considered when planning a service model.

In addition to the structure of the service and the type of counselling provided, pharmacotherapies offer important support to those quitters wishing to utilise them. Across all PCTs, NRT was most commonly prescribed (quit rate range 40.0-54.3%), followed by varenicline (quit rate range 60.2-81.0%), and then bupropion (quit rate range 37.5-90.9%). The quit rate range for quitters using counselling alone was 35.5-56.5% across the PCTs although it is likely that these are the most motivated patients and, although the data was not collected, it would be interesting to assess whether these were also clients attempting to quit for the first time.

Clearly there are limitations within this service evaluation dataset and in future work it would be useful to collect levels of deprivation and dependence and socio-economic factors to allow statistical analysis and further conclusions to be drawn as to the most appropriate service/intervention for specific client profiles. In addition, more robust data allowing for the comparison of different models of provision is needed to enable Stop Smoking Services to offer the most cost effective service for their local population.

## Conclusion

It is clear from the complexity of the results that quit rates associated with smoking cessation are multifactorial, with service structure, method of support delivery, healthcare professional involved and pharmacological support all playing a significant and important role in a quit attempt. The same models of service provision within different PCTs yield different quit rates. This is in line with recent research which reported substantial variation in success rates across intervention characteristics even after adjusting for smoker characteristics [35]. Clearly no single service will suit all and clients attempting to stop smoking need to be provided with support tailored to their individual needs. Patient choice and access to varied services is a key factor for consideration.

This evaluation has provided a useful benchmarking exercise, allowing for between-PCT descriptions of local service provision and patterns of service use to emerge, and highlighting areas for further investigation for potential service quality improvements. However, the findings need to be understood within the context of local health geography challenges and different client mixes. Stop Smoking Services with specialist knowledge of these factors are essential if smoking rates are to continue to be successfully reduced in future.

## Availability of supporting data

The data sets supporting the results of this article are held within the final evaluation report which is held by each author.

## Competing interests

Fran Percival is employed by pH Associates Ltd, which was under contract with Pfizer for the development, management and reporting of the service evaluation. Ian Lockhart is an employee of Pfizer. The other authors declare they have no competing interests.

## Authors' contributions

TM, SM and JW inputted into the evaluation design, sought and obtained Trust approval and oversaw the evaluation at each of their respective Trusts. They were also integral to the drafting of the manuscript. FP conceived of the evaluation, inputted into design and coordination and drafted the manuscript. IL inputted into the evaluation design and drafting of the manuscript. All authors read and approved the final manuscript.
